# Development of a recumbent isometric yoga program for patients with severe chronic fatigue syndrome/myalgic encephalomyelitis: A pilot study to assess feasibility and efficacy

**DOI:** 10.1186/s13030-017-0090-z

**Published:** 2017-03-03

**Authors:** Takakazu Oka, Hisako Wakita, Keishin Kimura

**Affiliations:** 1 Department of Psychosomatic Medicine, Graduate School of Medical Sciences, Kyushu University, Maidashi 3-1-1, Higashi-ku, Fukuoka, 812-8582 Japan; 2 Japan Yoga Therapy Society, Sanbonmatsu 1-2-24, Yonago, Tottori 683-0842 Japan

**Keywords:** Chronic fatigue syndrome (CSF), Isometric yoga, Fatigue, Treatment, Severe case, Myalgic encephalomyelitis (ME)

## Abstract

**Background:**

Our previous randomized controlled trial demonstrated that isometric yoga in a sitting position reduces fatigue in patients with chronic fatigue syndrome/myalgic encephalomyelitis (CFS/ME). However, some patients experience difficulties sitting or practicing isometric yoga in a sitting position for long periods. To date, therapeutic interventions for patients with severe symptoms have not been established. Therefore, we developed a recumbent isometric yoga program, which takes approximately 20 min to complete, designed to reduce fatigue in patients with severe CFS/ME. The aim of this pilot study was to assess the feasibility, safety, and usefulness of this program.

**Methods:**

This pilot study included 12 adult patients with CFS/ME. Six patients were reluctant to practice isometric yoga in a sitting position because of the severity of their fatigue (group 1). The remaining six patients had previously practiced isometric yoga in a sitting position (group 2). For 3 months, the patients of both groups practiced recumbent isometric yoga every 2 to 4 weeks with a yoga instructor and at home on other days if they could. The short-term effects of isometric yoga on fatigue were assessed using the Profile of Mood Status (POMS) questionnaire immediately before and after their final session with the yoga instructor. The long-term effects of isometric yoga on fatigue were assessed using the Chalder Fatigue Scale (FS) questionnaire before and after the intervention period. Adverse events, satisfaction with the program, and preference of yoga position (sitting or recumbent) were also recorded.

**Results:**

All subjects completed the intervention. In both groups, the POMS fatigue score was significantly decreased after practicing the 20-min yoga program and the Chalder FS score was decreased significantly after the 3-month intervention period. There were no serious adverse events. All subjects in group 2 preferred the recumbent isometric yoga program over a sitting yoga program.

**Conclusions:**

This study suggests that recumbent isometric yoga is a feasible and acceptable treatment for patients with CFS/ME, even for patients who experience difficulty practicing isometric yoga in the sitting position.

## Background

Chronic fatigue syndrome, or myalgic encephalomyelitis (CFS/ME), is a disorder characterized by persistent post-exertional fatigue and substantial symptoms related to cognitive, immune, and autonomic dysfunction [1, 2]. Currently, CFS/ME is treated with pharmacotherapy [3], cognitive behavioral therapy (CBT) [4, 5], and/or graded exercise therapy (GET) [6–8]. However, there are patients who do not show satisfactory improvement with these conventional treatments.

In a previous randomized controlled trial, we demonstrated that 2 months of practicing a daily 20-min program of isometric yoga in a sitting position relieved fatigue and pain in patients with CFS/ME who were resistant to conventional therapies [9]. Adult patients who could sit for more than 30 min were enrolled in an isometric yoga program and were instructed while sitting. Patients with more severe conditions who experienced difficulty sitting for 30 min or who spent most of their time in bed were not enrolled in the trial. Currently, there are no treatments established for patients with severe CFS/ME.

In order to increase participation in yoga, a psychosomatic medicine specialist (author TO) and two yoga therapists (authors HW and KK), discussed and developed a recumbent isometric yoga program that could be practiced by patients who spend most of their time in bed. The aim of this pilot study was to assess the feasibility and fatigue-relieving effect of recumbent isometric yoga in patients with severe CFS/ME.

## Methods

This study was approved by the Institutional Review Board of Kyushu University. Written informed consent was obtained from all study participants before they were enrolled.

### Subjects

Subjects were outpatients with CFS/ME who visited the Department of Psychosomatic Medicine of Kyushu University Hospital and satisfied the following criteria: (1) insufficient improvement of fatigue with treatments (previously described [10, 11]) including pharmacotherapy (antidepressants, Japanese traditional herbal medicine [12, 13], and/or coenzyme Q10), psychotherapy, GET, and/or autogenic training [11] for at least 6 months; and (2) between 20 and 70 years old. Patients were excluded if their fatigue was due to physical disease such as liver, kidney, heart, respiratory, endocrine, autoimmune, or malignant disease, severe anemia, electrolyte abnormalities, obesity, and/or pregnancy. The diagnosis of CFS/ME was made for patients who met the 1994 Fukuda definition of CFS [1] and the 2011 International Consensus Criteria [2].

Patients were allocated to one of two groups. Group 1 included six patients who had been excluded from our previous randomized controlled trial [9] because their symptoms were too severe and precluded them from practicing isometric yoga while sitting (ie, they spent more than 50% of the time when they were awake in bed). Group 2 included six patients who had previously practiced isometric yoga while sitting. Among them, three patients had been enrolled in the previous randomized controlled trial [9] and two continued to practice sitting isometric yoga after the conclusion of the trial.

### Level of function

Before starting this study, each patient’s level of function was assessed by two parameters: a performance status score based on the Japanese definition of CFS/ME [14] and a physical functioning subscale score based on the standard version of the Medical Outcomes Study Short Form 8, standard version (SF-8^TM^) [15]. First, attending physicians scored each patient’s performance status by rating their severity of fatigue and their level of functioning, which ranged from 0 (best performance status) to 9 (worst performance status) [14]. Second, patients completed the SF-8^TM^ and its physical functioning subscale scores were assessed.

### Development of the recumbent isometric yoga program

We discussed and developed a 20-min isometric yoga program that we anticipated could be practiced by patients who typically spend almost their whole day in bed, without exacerbating their symptoms or causing post-exertional malaise.

**Fig. 1:**
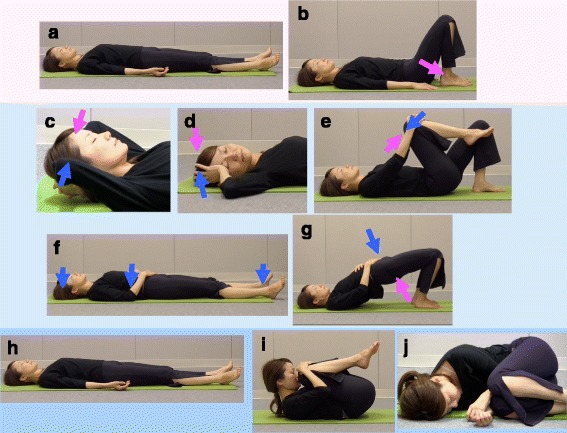
Illustration of poses from the recumbent isometric yoga program for CFS/ME. The 20-min recumbent isometric yoga program consists of three parts (I–III): I. Adjusting external and internal conditions. Awareness of body and breathing while in the recumbent position (**a**). Orientation of the body: relaxation of excessive lumbar lordosis (**b**). II. Isometric yoga. Isometric yoga for the neck (**c**). Isometric yoga for the neck and shoulder (**d**). Isometric yoga for the lower back and hip (**e**). Isometric yoga for the heels, elbows, and head (**f**). Loading and unloading of the hip (**g**). III. Deep relaxation and awakening. Sava-asana (**h**). Fetal pose (**i**). Relaxation in the lateral decubitus position (**j**)

The recumbent isometric yoga program we developed was designed to be performed on a bed and consists of three parts that included (1) adjusting external and internal conditions, (2) isometric yoga poses, and (3) deep relaxation and awakening. First, utmost attention was paid to external stimuli such as temperature, humidity, sound, smell, and light, so that patients in a recumbent position could practice isometric yoga in a comfortable environment with minimal stress. The room was equipped with brightness-adjustable ceiling lights that were adjusted by the instructor according to the preference of the recumbent patients before starting the session. The instructor remained mindful of the volume and tone of her voice. Patients were also asked to be mindful of these factors when they practice at home to help promote an environment in which deep relaxation could be facilitated. Second, patients were to practice isometric yoga poses as shown in Fig. 1. These poses were to be performed very slowly while breathing, with or without coordinated sounds, and with the patient using approximately 50% of their maximal muscular strength. Third, patients were required to completely relax and subsequently awaken. The session was designed to take approximately 20 min to complete.

Isometric yoga differs from traditional yoga postures in several ways, as has been described in more detail elsewhere [9]. The predominant difference is that the poses in this new program mainly require isometric muscle contraction. Because patients can modulate resistance depending on their level of fatigue, we hypothesized that isometric yoga would help prevent the exacerbation of fatigue. The isometric yoga poses do not include isotonic muscular contractions or active stretching and require less physical flexibility, which made them easier to practice while negating exacerbation of myalgia and/or arthralgia induced by overstretching. Similar to traditional yoga poses, isometric yoga poses are performed slowly with a mindfulness of one’s breath while synchronizing breathing and movement, as well as maintaining an awareness of inner sensations.

### Recumbent isometric yoga program

Here we describe the precise procedure of this program. Patients practiced recumbent isometric yoga on a yoga mat on the floor when they practiced it with a yoga instructor and on a tatami mat or on their bed when they practiced it at home.

I. *Adjusting external and internal conditions*
  (1) Environment: The yoga instructor strives to optimize the brightness of the lighting, the temperature, the tone of voice used, and the pace of the session to prioritize patient comfort and induce relaxation. Guidance is also provided on whether or not patients’ eyes should be closed. (2) Awareness of body and breathing while in the recumbent position: Patients should place their backs on the mat and relax while breathing naturally. They should be aware of themselves lying there. Attention should be focused on the consciousness of their own bodies, such that the patients feel different than they do when they normally lie down (Fig. 1a). (3) Orientation of the body: An understanding that it is possible to relax more deeply can help patients to prepare their body for the yoga session. First, they should feel that tension around their waist is relaxed by bending their knees. Hands are then placed on the side of the waist and the hips and buttocks are lifted slightly. The waist is relaxed while sliding the hips down towards the heels (Fig. 1b).

II. *Isometric yoga poses*
  During isometric loading, three repetitions are performed at about 50% of maximum muscle strength while exhaling and saying “umm” twice and remaining silent after the last repetition. After the isometric loading and while inhaling, patients should relax their muscles while maintaining their position. Once completely relaxed, a return to the first position is required while exhaling slowly, followed by the adoption of a relaxing pose (Sava-asana), that is, lying in a supine position with the arms and legs spread, the eyes closed, and breathing deeply. During the relaxation, changes in body sensation before and after applying the isometric load should be noted. While relaxing, the site affected by the isometric load should become slightly but noticeably warm. The amount of force, number of repetitions, and break between repetitions should be adjusted for the degree of fatigue experienced by the patient.
  (1) Isometric loading of the nape of the neck by pushing with both hands: the head should be lifted slightly before directing force towards the mat in the opposite direction to the resistance applied by the palms of the hands (Fig. 1c). (2) Isometric loading by rotating the neck to the left and right: While breathing in, the head should be slowly rotated to the right while exhaling, and that position is held as the right elbow is bent and the right palm is placed on the right temple. Force is then applied by the palm in the direction opposite that in which the head was rotated to provide resistance (Fig. 1d). (3) Isometric yoga for the lower back and hip: To generate isometric loading, pull the right knee with the hands towards the chest while simultaneously putting force into the knee as if to extend the leg. After applying the load, the force on the arms can be slowly relaxed so that the arms as well as the right foot can be lowered, and, with slow stretching, the legs should open and relax to the width of the shoulders. This can be repeated on the left side in the same manner (Fig. 1e). (4) Isometric yoga for heels, elbows, and head: Awareness of recumbent position: In a supine position, bend the elbows and place both palms on the belly; lift their elbows once before naturally returning them to a lower position and remain aware of the experience. Heel pressure in the mat or bed: While lightly pushing on the heels, force should be applied into the mat or bed. Elbow pressure on the mat: Both elbows should be used to apply force into the mat. Pressure on the mat from the back of the head: press the head into the mat (Fig. 1f). (5) Loading and unloading the hip: In the Sava-asana position, bend the elbows, place both palms on the stomach, bend both knees while the legs remain open, and bring the heels up towards the buttocks. After exhaling slowly, inhale while pressing lightly on the stomach with both palms, slowly lifting the waist, and then move the waist down while exhaling. Once down, the heels can be brought up towards the hips and buttocks (Fig. 1g).

**Fig. 2:**
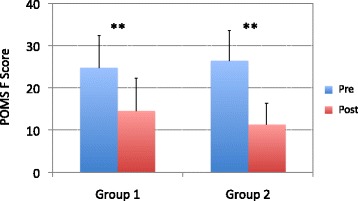
Short-term effects of recumbent isometric yoga on fatigue. The fatigue (F) scores from the Profile of Mood States (POMS) are compared before (pre, *blue*) and immediately after (post, *red*) the final 20-min session of the recumbent isometric yoga program with the instructor and patients from group 1 (*n* = 6) and group 2 (*n* = 6). ** *P* = 0.01 (paired *t* test)

**Fig. 3:**
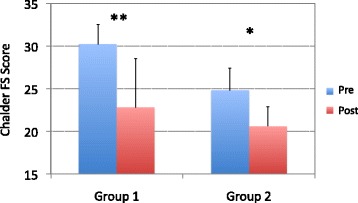
Long-term effects of recumbent isometric yoga on fatigue. The Chalder Fatigue Scale (FS) scores are compared before (pre, *blue*) and after (post, *red*) the intervention period in patients from group 1 (*n* = 6) and group 2 (*n* = 6). ** *P* < 0.01, * *P* < 0.05 (paired-sample *t* test)

III. *Deep relaxation and awakening*
  (1) Sava-asana (Fig. 1h), (2) fetal pose (Fig. 1i), (3) relaxation in the lateral decubitus position (Fig. 1j), and (4) awakening.

### Yoga intervention

Patients with CFS/ME were asked to practice recumbent isometric yoga using the same 20-min program for 3 months. During the intervention period, if pharmacotherapy had been previously prescribed, it was continued and medication dosages were not changed. On the day patients visited the hospital, they practiced recumbent isometric yoga for 20 min on a one-on-one basis with an instructor. The instructor had more than 30 years of experience as a yoga instructor and had more than 3 years of experience instructing patients with CFS/ME in isometric yoga techniques. Lessons were given between 2 pm and 4 pm. During the yoga program, the instructor was not allowed to play background music, which is often used in yoga studios to facilitate the relaxation of participants. Before and after practicing isometric yoga, the supervising doctor checked their patient’s condition and recorded any adverse events or any uncomfortable symptoms, such as exacerbation of fatigue, pain, dizziness, or anxiety that could possibly be caused by practicing isometric yoga. In addition to receiving a private lesson, the patients were asked to practice this program outside of the lessons provided if it was possible, with the aid of a digital videodisc and a booklet of the recumbent isometric yoga program.

### Assessment of outcomes

1. *Short*- *and long*-*term effects of recumbent isometric yoga on fatigue*
  To assess the short-term effect of recumbent isometric yoga on fatigue, the fatigue (F) scale scores of the Profile of Mood States (POMS) [16], a self-rating questionnaire, were compared immediately before and after the final 20-min session of isometric yoga with the instructor. To assess the long-term effects of yoga on fatigue, the 11-item Chalder fatigue scale (FS) [17] was used; it is a well-validated, self-reported scale for measuring the severity of fatigue experienced by patients with CFS/ME. Scores were compared before and after the 3-month intervention period. The Chalder FS questions were deemed inappropriate for evaluating short-term changes in fatigue.

2. *Feasibility*
  Feasibility was assessed by evaluating the retention rates, adherence, adverse events, patients’ self-reported levels of satisfaction, and their preference for recumbent isometric yoga over isometric yoga in a sitting position. Adverse events were monitored in two ways. First, at each visit to the hospital the supervising doctor recorded any discomfort or symptoms reported by the patient after practicing yoga with the instructor. Second, patients were asked to keep a yoga diary and record the amount of time they practiced the isometric yoga and to document how they felt after practicing it. On the day of the visit to the hospital, the supervising doctor reviewed the diary and determined if the patient had experienced any symptoms of discomfort. After the intervention period, the diary was collected to determine how often the subjects had practiced yoga at home. On the day the patients practiced recumbent isometric yoga with an instructor for the final time, the supervising doctor asked the patients in group 2 about their preferred yoga program. Preference was evaluated in a consultation room where the yoga instructor was not present and by just asking which isometric yoga program was preferred—the sitting version or recumbent version, or was there no preference?—and the reasons for their preference.

### Statistical analyses

The data are presented as the mean ± SD. The differences in the POMS-F and Chalder FS scores measured before and after the intervention were tested using a paired-sample *t* test. Between-group differences in age and in the POMS-F, Chalder FS, and SF-8 physical functioning subscale scores were tested using an independent-sample *t* test. Two-tailed tests were used. Data were analyzed by using SPSS for Windows, V.17.

## Results

### Patients

The study comprised 12 patients (age range: 26–61 years; mean age: 39.5 ± 11.0 years; 10 women and 2 men) divided equally among group 1 (age range: 26–54 years; mean age: 34.8 ± 10.3 years; 6 women) and group 2 (age range: 29–61 years; mean age: 44.2 ± 12.5 years; 4 women and 2 men). Mean age was not significantly different between groups. The baseline Chalder FS score was higher for the patients in group 1 (30.2 ± 2.3) than for those in group 2 (24.8 ± 2.6) (*P* < 0.01). The performance status of all patients in group 1 was 7, which meant they were all unable to carry on normal activity or perform light tasks; however, they were able to care for themselves without assistance. By comparison, in group 2 there were three patients with a performance status of 6, indicating they required rest at home without working for more than one-half of a week. Another three patients in group 2 were performance status 5, incapable of carrying on normal activity or doing any active work except light tasks; moreover, they required rest at home without work for several days a week. In contrast, SF-8 physical functioning subscale scores were not significantly different between group 1 (28.1 ± 7.9) and group 2 (37.9 ± 8.4) (*P* = 0.07) (Table 1).

**Table 1:** Demographic characteristics of the patients

	Total	Group 1	Group 2
Number (female:male)	12 (10:2)	6 (6:0)	6 (4:2)
Age (years)	39.5 ± 11.0	34.8 ± 10.3	44.2 ± 12.5
Chalder FS score **	27.5 ± 3.6	30.2 ± 2.3	24.8 ± 2.6
Performance status (number)		5 (0)	5 (3)
		6 (0)	6 (3)
		7 (6)	7 (0)
SF-8 physical functioning	33.0 ± 9.3	28.1 ± 7.9	37.9 ± 8.4

Data are the mean ± standard deviation. Number in the parenthesis represents the number of patients

** *P* < 0.01 by an independent-sample *t* test (group 1 vs group 2)

Most patients visited their doctor every 2 to 4 weeks during the intervention period. Therefore, patients practiced the same isometric yoga program at least four times with the instructor during the intervention period. They mostly practiced the same 20-min program; however, some patients decreased the number of repetitions per pose depending on their physical condition. It took some patients 30 min to complete the program because they performed the poses more slowly, took a longer than usual break in between poses, and/or relaxed longer during poses.

### Short-term effect of recumbent isometric yoga on fatigue

The POMS-F score was compared immediately before and after the final session of isometric yoga with an instructor. The POMS-F score before the session was not different between group 1 and group 2. After the session, the POMS-F score significantly decreased relative to the score obtained immediately before the final instructor-led session of isometric yoga in both group 1 (24.7 ± 7.6 to 14.5 ± 7.8, *P* = 0.01) and group 2 (26.3 ± 7.2 to 11.3 ± 5.0, *P* = 0.01). Short-term effects of recumbent isometric yoga on fatigue, measured by changes in the POMS-F score between immediately before and after the final session of isometric yoga with an instructor, were not different between group 1 (10.2 ± 6.2) and group 2 (15.0 ± 9.3) (*P* = 0.32) (Fig. 2).

### Long-term changes in fatigue scores

Chalder FS scores significantly decreased from baseline to the final instructor-led isometric yoga session at 3 months in group 1 (30.2 ± 2.3 to 22.8 ± 5.7, *P* < 0.01) and group 2 (24.8 ± 2.6 to 20.7 ± 2.3, *P* < 0.05). The long-term effect of recumbent isometric yoga on fatigue, measured by change in Chalder FS score between baseline and the final instructor-led isometric yoga session at 3 months, was not different between group 1 (7.3 ± 4.3) and group 2 (4.2 ± 3.8) (*P* = 0.23) (Fig. 3).

### Feasibility

#### Retention rates

All patients completed the intervention; no patients discontinued the study.

#### Adherence

Overall adherence was very good. All patients practiced yoga with an instructor when they visited the hospital. Seven out of 12 patients (58%) kept yoga diaries. Based on those diary records, recumbent isometric yoga was practiced at home on average 6.0 ± 0.8 days/week and 5.6 ± 1.0 days/week during the first and last weeks of the intervention period, respectively.

#### Safety

No patients reported any adverse symptoms, including post-exertional malaise. One patient reported that she felt extremely tense when she heard the word “fetal” poses, which reminded her of a psychologically traumatic memory in her early life, therefore, we refrained from using the word. Thereafter, she had no difficulty practicing recumbent isometric yoga.

#### Satisfaction

All patients reported a high level of satisfaction with the program and described recumbent isometric yoga as being useful and helpful. Preferences: All subjects in group 2 preferred isometric yoga in a recumbent position over a sitting position and thought it was comparatively more useful for the following reasons: (1) less energy was required and it was easier to do, (2) more relaxation and comfort were experienced, (3) it was possible to practice on days when sitting was problematic, such as on days when symptoms were less tolerable, and (4) the program could be practiced on a bed, which was a most important consideration.

## Discussion

This study demonstrated that recumbent isometric yoga was associated with decrease in POMS-F scores and Chalder FS scores in patients with CFS/ME who had not improved satisfactorily after at least 6 months of conventional treatment. Patients in group 1 included those whose fatigue level was too severe to practice isometric yoga while sitting. Therefore, it is reasonable that the Chalder FS score in group 1 was higher than that in group 2. However, reduced POMS-F and Chalder FS scores were associated with the practice of isometric yoga in both groups. Therefore, the present findings suggest that a single session of recumbent isometric yoga can reduce fatigue in patients with severe CFS/ME who are accustomed to the yoga procedures. Furthermore, this study also suggests the possibility that regular practice of recumbent isometric yoga has a long-term fatigue-relieving effect. With no control group in this study, this possibility is speculative and must be reevaluated by a randomized, controlled trial.

This study also suggests that recumbent isometric yoga is a feasible treatment modality for CFS/ME. Previous studies have demonstrated that some patients with CFS/ME experience adverse events such as worsening of fatigue and deterioration of physical function when treated with conventional therapies such as CBT, GET, or specialist medical care, although the incidence of events did not differ among treatments [18]. It is also possible for yoga to cause unfavorable symptoms and adverse events [19, 20]. Therefore, we carefully developed this program so as not to exacerbate the symptoms of patients, and especially to reduce the risk of inducing post-exertional malaise. The results demonstrated that patients had no serious adverse events and no post-exertion malaise.

Patients also showed an excellent level of adherence and were highly satisfied with the program. Furthermore, all patients in group 2 preferred the recumbent isometric yoga program to a sitting one. Their reasons appeared logical, as it was a treatment for severe CFS. For example, patients reported that they could practice a recumbent yoga program even when they were not physically able to practice a program while sitting. Furthermore, they also reported feeling deeper relaxation because less energy was expended by isometric yoga in a recumbent compared with in a sitting position. Taken together, this pilot study suggests that a recumbent isometric yoga program is both feasible and effective for patients with severe CFS/ME.

When we developed this program, we considered the following characteristics of patients with severe CFS/ME. Although they spend most of their time in bed, some patients do not necessarily rest in a truly relaxing recumbent position. Therefore, we began by aiming to make those patients feel more relaxed and comfortable by helping them properly adjust their recumbent postures. For example, in some patients, excessive lumbar lordosis was observed, which may increase muscular tension in the back and prevent relaxation when lying on a bed. Therefore, we instructed the patients to be aware of this and we provided guidance by informing them that they could lessen the excessive lordosis in their lumbar spine. We further instructed the patients to focus on feeling the changes associated with the relaxation of their muscular tension induced by the adjustments they achieve through yoga. When the patients rested in Sava-asana, we also instructed them about the positioning of their scapulae and arms so that they could breathe more easily and deeply. For many patients, these instructions helped them lay in a more relaxed and comfortable position. After these instructions (part 1 of this program), we provided instruction on isometric yoga for the neck and chest to relax the respiratory support muscles (part 2). These changes, at least in part, might contribute to the short-term fatigue-relieving effect of recumbent isometric yoga.

In order to achieve long-term fatigue-relieving effects, several mechanisms needed to be considered. First, patients with CFS/ME tend to be physically deconditioned [21, 22]. Physical deconditioning is suggested to contribute to the fatigue associated with CFS/ME [23]. For this reason, the yoga program was designed to act as an exercise therapy to reverse deconditioning and it included isometric exercises for the antigravity muscles. For patients with severe CFS/ME, aerobic exercise is sometimes too difficult to perform, whereas the present study demonstrated that recumbent isometric yoga seems to be tolerable and an acceptable way for them to get some exercise. Second, autonomic disturbance resulting in sympathetic overactivity and low vagal tone is also suggested to be involved in CFS/ME [24, 25]. Generally, an isometric muscular contraction is associated with activation of sympathetic nerves and an increase in blood pressure (BP) [25–27]. However, regular practice of isometric exercise for several months decreases BP and can improve cardiac parasympathetic function [28–31]. Therefore, isometric yoga could potentially reverse deconditioning and improve vagal function, thereby mitigating fatigue in the long-term; however, future studies are necessary to validate this possibility. Third, hypofunction of the hypothalamic-pituitary-adrenocortical axis and immune dysfunction, characterized by increased proinflammatory cytokines and inflammatory parameters, are also reported to be associated with CFS/ME [10, 32]. However, at present it is not known whether recumbent isometric yoga modulates these abnormalities.

In this isometric yoga program, we instructed the patients to be aware of inner sensations known to occur with traditional yoga instruction. One may question whether interventions that can shift a patient’s attention from outer world stimuli to inner sensations, especially proprioception, can exacerbate feelings of fatigue. Without any other instructions beyond paying attention to inner sensations, that could be the case. However, in this program, when we asked the patients to focus on inner sensations, we also provided instruction on body position and asked them to practice isometric yoga. As was reflected by the changes in the POMS-F scores, these instructions and the practice they did were associated with a reduction in fatigue. This was achieved by directing attention to sensations related to changing to a more comfortable position, being more relaxed, peaceful, and warmer, such that feelings of fatigue were not the focus. Thus, focusing on proprioception became meaningful rather than distressing; therefore, inner sensations provided beneficial effects.

This study had several limitations. First, the patients received pharmacotherapy concurrent with practicing isometric yoga during the intervention period. To minimize pharmacotherapy-induced changes in fatigue, we selected patients who had not exhibited satisfactory improvement in their symptoms after 6 months of conventional therapy, which included pharmacotherapy. We were able to show that a single session of recumbent isometric yoga can reduce the POMS-F score. However, there remains a possibility that the long-term fatigue-relieving effect, which was shown by changes in the Chalder FS score, was not induced by isometric yoga alone, but by the sum or the synergistic effect of pharmacotherapy and isometric yoga. Second, although this study indicated that recumbent isometric yoga could reduce fatigue in patients with CFS/ME, the mechanisms underlying the fatigue-relieving effects are not clear. We have suggested possible mechanisms as discussed above, but they remain speculative at this time. Third, because this was a pilot study to investigate the feasibility of the recumbent isometric yoga program for severe CFS/ME patients, the number of subjects (*n* = 12) was relatively small. Fourth, changes in the functional level of patients over the intervention period were not assessed in this study.

Based on weighing the present promising results against the shortcomings, we are currently conducting a randomized, controlled trial to assess the effectiveness of recumbent isometric yoga in patients with severe cases of CFS/ME and plan to further explore the possible underlying mechanisms by which improvements in symptoms occur.

## Conclusions

This study suggests that recumbent isometric yoga is a feasible and acceptable treatment for patients with CFS/ME, including those who experience difficulties practicing isometric yoga while sitting and those who spend almost all day in bed. Further studies are needed to determine the effectiveness of recumbent isometric yoga.

## Abbreviations

BP: Blood pressure
CBT: Cognitive behavioral therapy
CFS: Chronic fatigue syndrome
F: Fatigue
FS: Fatigue scale
GET: Graded exercise therapy
ME: Myalgic encephalomyelitis
POMS: Profile of mood states
SD: Standard deviation
SF-8: Short form-8

## References

[1] Fukuda K, Straus SE, Hickie I, Sharpe MC, Dobbins JG, Komaroff A (1994). The chronic fatigue syndrome: a comprehensive approach to its definition and study. International Chronic Fatigue Syndrome Study Group. Ann Intern Med.

[2] Carruthers BM, van de Sande MI, De Meirleir KL, Klimas NG, Broderick G, Mitchell T, Staines D, Powles AC, Speight N, Vallings R (2011). Myalgic encephalomyelitis: International Consensus Criteria. J Intern Med.

[3] Collatz A, Johnston SC, Staines DR, Marshall-Gradisnik SM (2016). A systematic review of drug therapies for chronic fatigue syndrome/myalgic encephalomyelitis. Clin Ther.

[4] Price JR, Mitchell E, Tidy E, Hunot V. Cognitive behaviour therapy for chronic fatigue syndrome in adults. Cochrane Database Syst Rev. 2008;(3):CD001027. doi:10.1002/14651858.CD001027.pub2 10.1002/14651858.CD001027.pub2 PMC7028002 18646067

[5] Wiborg JF, van Bussel J, van Dijk A, Bleijenberg G, Knoop H (2015). Randomised controlled trial of cognitive behaviour therapy delivered in groups of patients with chronic fatigue syndrome. Psychother Psychosom.

[6] White PD, Goldsmith KA, Johnson AL, Potts L, Walwyn R, DeCesare JC, Baber HL, Burgess M, Clark LV, Cox DL (2011). Comparison of adaptive pacing therapy, cognitive behaviour therapy, graded exercise therapy, and specialist medical care for chronic fatigue syndrome (PACE): a randomised trial. Lancet.

[7] Chalder T, Goldsmith KA, White PD, Sharpe M, Pickles AR (2015). Rehabilitative therapies for chronic fatigue syndrome: a secondary mediation analysis of the PACE trial. Lancet Psychiatry.

[8] Larun L, Brurberg KG, Odgaard-Jensen J, Price JR (2016). Exercise therapy for chronic fatigue syndrome. Cochrane Database Syst Rev.

[9] Oka T, Tanahashi T, Chijiwa T, Lkhagvasuren B, Sudo N, Oka K (2014). Isometric yoga improves the fatigue and pain of patients with chronic fatigue syndrome who are resistant to conventional therapy: a randomized, controlled trial. Biopsychosoc Med.

[10] Oka T, Kanemitsu Y, Sudo N, Hayashi H, Oka K (2013). Psychological stress contributed to the development of low-grade fever in a patient with chronic fatigue syndrome: a case report. Biopsychosoc Med.

[11] Oka T (2015). Efficacy and limitations of autogenic training as a treatment of chronic fatigue syndrome. Jpn J Autogenic Ther.

[12] Okumi H, Koyama A (2014). Kampo medicine for palliative care in Japan. Biopsychosoc Med.

[13] Oka T, Okumi H, Nishida S, Ito T, Morikiyo S, Kimura Y, Murakami M (2014). Effects of Kampo on functional gastrointestinal disorders. Biopsychosoc Med.

[14] Matsuda Y, Matsui T, Kataoka K, Fukada R, Fukuda S, Kuratsune H, Tajima S, Yamaguti K, Kato YH, Kiriike N (2009). A two-year follow-up study of chronic fatigue syndrome comorbid with psychiatric disorders. Psychiatry Clin Neurosci.

[15] Fukuhara S, Suzukamo Y (2004). Mannual of the SF-8 Japanese version.

[16] McNair D, Lorr M, Droppleman L (1971). Mannual for the Profile of Mood States (POMS).

[17] Chalder T, Berelowitz G, Pawlikowska T, Watts L, Wessely S, Wright D, Wallace EP (1993). Development of a fatigue scale. J Psychosom Res.

[18] Dougall D, Johnson A, Goldsmith K, Sharpe M, Angus B, Chalder T, White P (2014). Adverse events and deterioration reported by participants in the PACE trial of therapies for chronic fatigue syndrome. J Psychosom Res.

[19] Cramer H, Ward L, Saper R, Fishbein D, Dobos G, Lauche R (2015). The safety of yoga: A systematic review and meta-analysis of randomized controlled trials. Am J Epidemiol.

[20] Matsushita T, Oka T (2015). A large-scale survey of adverse events experienced in yoga classes. Biopsychosoc Med.

[21] Fulcher KY, White PD (2000). Strength and physiological response to exercise in patients with chronic fatigue syndrome. J Neurol Neurosurg Psychiatry.

[22] Nijs J, Aelbrecht S, Meeus M, Van Oosterwijck J, Zinzen E, Clarys P (2011). Tired of being inactive: a systematic literature review of physical activity, physiological exercise capacity and muscle strength in patients with chronic fatigue syndrome. Disabil Rehabil.

[23] Riley MS, O’Brien CJ, McCluskey DR, Bell NP, Nicholls DP (1990). Aerobic work capacity in patients with chronic fatigue syndrome. BMJ.

[24] Boneva RS, Decker MJ, Maloney EM, Lin JM, Jones JF, Helgason HG, Heim CM, Rye DB, Reeves WC (2007). Higher heart rate and reduced heart rate variability persist during sleep in chronic fatigue syndrome: a population-based study. Auton Neurosci.

[25] Wyller VB, Barbieri R, Thaulow E, Saul JP (2008). Enhanced vagal withdrawal during mild orthostatic stress in adolescents with chronic fatigue. Ann Noninvasive Electrocardiol.

[26] Araújo CG, Duarte CV, Gonçalves Fde A, Medeiros HB, Lemos FA, Gouvêa AL (2011). Hemodynamic responses to an isometric handgrip training protocol. Arq Bras Cardiol.

[27] Boulton D, Taylor CE, Macefield VG, Green S (2016). Contributions of central command and muscle feedback to sympathetic nerve activity in contracting human skeletal muscle. Front Physiol.

[28] Millar PJ, Bray SR, MacDonald MJ, McCartney N (2008). The hypotensive effects of isometric handgrip training using an inexpensive spring handgrip training device. J Cardiopulm Rehabil Prev.

[29] Kelley GA, Kelley KS (2010). Isometric handgrip exercise and resting blood pressure: a meta-analysis of randomized controlled trials. J Hypertens.

[30] Kanaley JA, Goulopoulou S, Franklin R, Baynard T, Carhart RL, Weinstock RS, Fernhall B (2012). Exercise training improves hemodynamic recovery to isometric exercise in obese men with type 2 diabetes but not in obese women. Metabolism.

[31] Millar PJ, Levy AS, McGowan CL, McCartney N, MacDonald MJ (2013). Isometric handgrip training lowers blood pressure and increases heart rate complexity in medicated hypertensive patients. Scand J Med Sci Sports.

[32] Klimas NG, Broderick G, Fletcher MA (2012). Biomarkers for chronic fatigue. Brain Behav Immun.

